# Binding of long-chain α-neurotoxin would stabilize the resting state of nAChR: A comparative study with α-conotoxin

**DOI:** 10.1186/1742-4682-6-3

**Published:** 2009-02-11

**Authors:** Adak Nasiripourdori, Bijan Ranjbar, Hossein Naderi-Manesh

**Affiliations:** 1 Department of Biophysics, Faculty of Science, Tarbiat Modares University, P.O. Box 14115-175, Tehran, Iran

## Abstract

**Background:**

The details of interaction in a complex between potent antagonists such as long chain α-neurotoxins and α-conotoxins with nicotinic acetylcholine receptor (nAChR), and conformational changes induced by these antagonists, are not yet clear.

**Modeling:**

In order to uncover some of these critical structural features, we conducted a docking simulation and a molecular dynamics simulation (MD) of a model of the ligand binding domain of nAChR in complex with a long-chain α-neurotoxin and an α-conotoxin.

**Results:**

Our docking results confirm the claim that *T*.nAChR is in the basal or resting state, which favors binding to the alpha-neurotoxins. Moreover, more correct "hits" for the α/γ interface upon docking for conotoxin-nAChR confirm the preference of conotoxin GI for the α/γ interface. More importantly, upon binding of α-neurotoxin, ligand-bonded nAChR is less dynamic in certain domains than the apo form of the conotoxin-AChR complex. Some critical interactions in the binding site such as the salt bridge formed between K145/D200 in the neurotoxin-nAChR complex is further stabilized during the MD simulation, while it is obviously more labile in the apo form.

**Conclusion:**

These observations could support the claim that alpha neurotoxins stabilize the nAChR resting state.

## Background

The nicotinic acetylcholine receptor (nAChR) is a member of the Cys-loop superfamily of ligand-gated ion channels (LGIC), which includes the neuronal acetylcholine receptor, the γ-aminobutyric acid receptor (GABA_A_R), the serotonin type 3 receptors (5-HT_3_R) and glycine receptors (GlyR) [1,2]. In general, AChRs can be divided into two main families: muscle type and neuronal nAChRs [3]. The muscle type is a heteropentamer consisting of α_1_, β_1_, δ and γ or ε subunits in the stochiometries (α_1_)_2_β_1_γδ or (α_1_)_2_β_1_εδ in embryonic or adult receptors, respectively. The extracelluar amino-terminal domain of nAChRs is approximately 210 amino acid residues long and contains binding sites for agonists and competitive antagonists located at the α-γ and α-δ subunit interfaces [4,5]. These two binding sites are non-equivalent and competitive antagonists show different affinities for them [5]. When a ligand binds, a chain reaction of conformational changes begins in the ligand binding domain (LBD) that is transmitted to the transmembrane domain (TMD), resulting in either an open form that allows the passage of ions across the channel, or a closed form that does not.

Several classes of ligands bind to nAChR. These include small molecules such as the endogenous agonists acetylcholine and carbamylcholine, small peptides including the lophotoxins and α-conotoxins (CTx), and large peptide toxins isolated from snake venoms (e.g., α-bungarotoxin, α-cobratoxins). α-Conotoxins that are specific for muscle AChRs include MI, GI and SI, and contain three residues in the first loop and five in the second. Muscle-specific α-conotoxins can be further subdivided according to their ability to select between the two AChR binding sites: MI and GI show 10,000-fold differences between the two binding sites, whereas CTx SI shows a 100-fold difference [6-8]. Because of their marked selectivity for the peripheral and neuronal forms of the receptor, as well as discrimination between nAChR binding sites, these toxins are valuable for probing structure-function relationships in various nAChR subtypes. The α-conotoxins are 12–18 residues long and are characterized by the presence of two conserved disulfide bonds and two loops in the peptide backbone between the cysteines. These small peptides are known to distinguish between the two antagonist binding sites of nAChRs [9], for example d-tubocurarine selectively blocks the interactions of α-conotoxins GI and MI with their higher affinity binding site on *Torpedo *receptors, suggesting that they have a higher affinity for the acetylcholine binding site near the α/γ subunit interface [8,10]. This is also consistent with another important class of nAChR antagonists, α-neurotoxins from snake venom, which utilize a common binding core consisting of key invariant residues to interact with subtype-specific receptor residues. The evidence for this comes from various mutational analyses, photoaffinity labeling data, NMR and X-ray studies [11,12]. α-Neurotoxins from *Elapid *and *Hydrophiid *snake venoms belong to the three-finger toxin superfamily of polypeptides containing 60–74 amino acid residues. The characteristic feature of all three-finger toxins is their distinctive structure, formed by three adjacent loops that emerge from a small, globular, hydrophobic core that is cross-linked by four conserved disulfide bridges [12].

It has long been known that the muscle-type nAChR undergoes conformational transitions between "basal" (resting), desensitized (closed) and open (active) channel states, each with distinctive affinities for acetylcholine [13]; and it has been proposed that α-neurotoxins stabilize the resting state of nAChR [14]. However, despite extensive studies on the gating mechanism of nAChR when it binds small agonists such as ACh, and molecular dynamics studies on the structural motions of homomeric α7 nAChR [15-17], there have been few in-silico studies of the dynamics and interaction of larger antagonists with nAChR. Because of the size of these receptors (~290 kDa), the only direct structure determinations have been at medium resolution (~4 Å) using electron microscopy [3]. Nicotinic acetylcholine receptors have been implicated in the pathophysiology of several neuropsychiatric disorders, including schizophrenia, Alzheimer's disease, Parkinson's disease, and Tourette's syndrome [18]; the development of clinically and experimentally useful drugs depends in part on determining the structural features of a ligand that contribute to its affinity and specificity. Therefore, more detailed structural analyses of nAChRs and their complexes with agonists and antagonists would be useful for designing more specific ligands and drugs.

In the current study, the binding mode of *T*.nAChR with two antagonists, an α-conotoxin and a long-chain α-neurotoxin, was determined. Also, the structural dynamics of the LBD of *T*.nAChR were examined during a nanosecond-scale molecular dynamics simulation without the ligand (apo form) and in the presence of each of the two antagonists. The major goal was to observe and compare the conformational changes in the LBD in the presence of these antagonists over this time period, and to examine the structural determinants that govern the binding of two inherently potent antagonists of different sizes. The models are based on the extracellular domain of *Torpedo *marmorata (2BG9) nAChR, which appears to exist in the basal or resting state (a suitable conformation for binding to α-neurotoxins) in complex with a long-chain α-neurotoxin from *Naja Naja oxiana *cobra venom (PDB accession number:1W6B) [20] and an α-conotoxin from *C. geographus *(PDB accession number:1XGA).

## Results and discussion

### An overall view of the nAChR complexes with antagonists

#### nAChR in complex with long chain neurotoxin NTX-1

**Figure 1:**
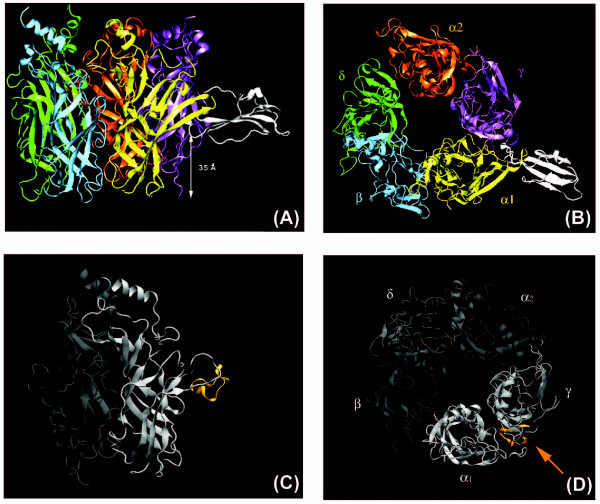
**Binding mode of NTX-1 with ligand binding domain of *Torpedo *nAChR from front (A) and top (B) view**. (C) and (D): Binding mode of 1XGA with ligand binding domain of *Torpedo *nAChR from front and top view, respectively.

According to our model, NTX-1 in complex with *T*.nAChR is located about 35 Ǻ from the membrane surface (Fig. 1A–B). The tip of the toxin central loop plugs deeply into the receptor, at the interfaces formed between two subunits: alpha_1 _with gamma, and alpha_2 _with delta (not shown here). The toxin lies almost equatorially to the extracellular domain of the nAChR, as previously suggested for the interaction of short- and long-chain α-neurotoxins with nAChRs [4,21-25] with its concave side facing the α_1 _subunit and loops I and III oriented towards the top and bottom of the receptor respectively (see Fig. 1A). The toxin molecular axis, defined by its central three antiparallel beta sheets, lies at ~85° relative to the main and ~80° to the median axis of the receptor. The tip of the toxin central loop II is positioned slightly under the C-loop in a small cavity located in the interface between the α_1 _and γ subunits and makes several contacts with functional loops from the principal (α_1_) and complementary (γ or δ) subunits. The only difference lies in the angle of the toxin axis relative to the main (α) and complementary (γ) binding sites, which is slightly different from the previously-reported Cbtx-AChBP complex [26]. Absence of a membrane lipid bilayer and the long dynamic C-tail of the toxin would inevitably affect the binding mode. Table 1 shows the main participating residues from toxin NTX-1, making contacts with the main (alpha) and complementary (gamma) binding sites from the initial complex obtained after docking (other amino acid residues participating in the interactions of NTX-1 with the receptor (α-γ) after the 7 ns MD simulation are shown [see Supplementary table 3 in additional file 1]. These anchor points have been reported to be the main interacting areas in long-chain α-neurotoxins [21,26-31]. Various experimental studies have shown that receptor fragment 184–200 from the α_1 _subunit (corresponding to the C-loop) is important for long-chain α-neurotoxin binding [11,23,26,29,32], where residues K185, W187, Y189, Y190, T191, C192, P194, Y198 and W149 have been reported to interact directly with α-bungarotoxin and α-cobratoxin [11]. In addition, residues W55, L119 and E176 from the complementary binding site (gamma subunit) are reported to interact mainly with the central loop of long-chain α-neurotoxins (loop II) [23]. These residues have been shown to be highly conserved among all species of nAChR to allow proper binding of agonists such as Ach and competitive antagonists through cation-p and hydrophobic interactions [11,33]. The functionally important residues for the *Torpedo *receptor are all located on the concave surface of short-chain and long-chain α-neurotoxins with the most critical residue, Arg35 (Arg33 in α-cobratoxin), located at the tip of loop II [12]. As can be seen in table 1 [and table 3 additional file 1], Arg 35 (corresponding to Arg33 and Arg36 in α-cbtx and α-bgtx, respectively) located at the tip of loop II in the toxin is surrounded by the aromatic residues Trp149, Thr150 (loop B), Tyr190 (loop C) Tyr93 (loop A) from the alpha_1 _and leu118, Pro 119 (loop E), Trp54, Glu56 (loop D), Glu175, Asp 176 (loop F) from the gamma subunits, which is in good agreement with previously reported complexes. It has been proposed that α-neurotoxins compete with acetylcholine by introducing the positive charge of Arg35 into the ligand-binding pocket of the receptor [12]. This residue makes its main hydrophobic contacts with Trp149, Asn94, Tyr93 and Tyr190 from the alpha_1 _and leu118, Trp54 from the gamma subunit (see table 1).

**Table 1:** Amino acid residues participating in interactions of NTX-1 with receptor interface determined by docking simulation.

	***T*.nAChR subunit interface**		
	
**NTX-1**	Principal site (alpha 1)	Complementary site (gamma)	Average occupancy during MD
Loop I			
Thr 6	Thr 191	Arg 186	
Ile 8	Pro 194, Cys 193, Cys 192, Thr 191	Ile 172	Ile8 Thr 191 (α1) 0.8
			Ile8 pro 194 (α1) 0.7
			Ile8 Cys 192 (α1) 0.9
			
Loop II			
TRp 31	Tyr 190, Val 188	Asp 176, Glu 175,	Trp31 Val 188 (α1) 0.9
			Trp31 Tyr 190 (α1) 0.9
Cys 32		Asp 176, Thr 37	Cys32 Asp 176 (γ) 0.9
			Cys32 Glu 175 (γ) 0.9
Gly 33		Trp 54	Gly33 Asp 176 (γ) 0.9
Ser 34	Tyr 93	Leu 118, Trp 54,	Ser34 Trp 54 (γ) 0.7
			Ser34 Tyr 93 (α1) 0.6
Arg 35*		Glu 175	Arg 35* Leu 118 (γ) 0.9
			Arg 35* Trp 54 (γ) 0.9
			Arg 35* Trp 149 (α) 0.9
Gly 36	Tyr 190	Gln 58	
Lys 37			Lys 37 Glu (γ) 0.8
Val 38	Thr 191		Val38 Thr 191 (α1) 0.9
Ile 39	Thr 191, Cys 192		Ile Thr 191 (α1) 0.9
			
Loop III			
Ser 52			
Tyr 53			Tyr53 Leu 172 (γ) 0.9
			
C-Tail			
Gln 70	Pro 194, Cys 193		Arg72 Cys 193 (α1) 1
Lys 71	Cys 193,		Lys71 Cys 193 (α1) 1
Arg 72	Cys 193		Gln70 Cys 193 (α1) 1
Pro 73	Cys 192, Cys 193		Gln70 Pro 194 (α1) 0.9

Average occupancies of the main anchor point residues participating in interactions of NTX-1 with receptor (α/γ interface) after 7 ns molecular dynamics simulation and within 5 Å distance along the simulation trajectory.The critical residue Arg 35 at the tip of Loop II is marked with a star. Amino acids making hydrogen bonds are underlined.

In summary, our complex model is in good agreement with earlier predictions [19], suggesting that long chain alpha-neurotoxin NTX-1 interacts similarly in many ways to other long-chain α-neurotoxins in binding to nAChR.

#### nAChR in complex with alpha-conotoxin 1XGA

**Figure 2:**
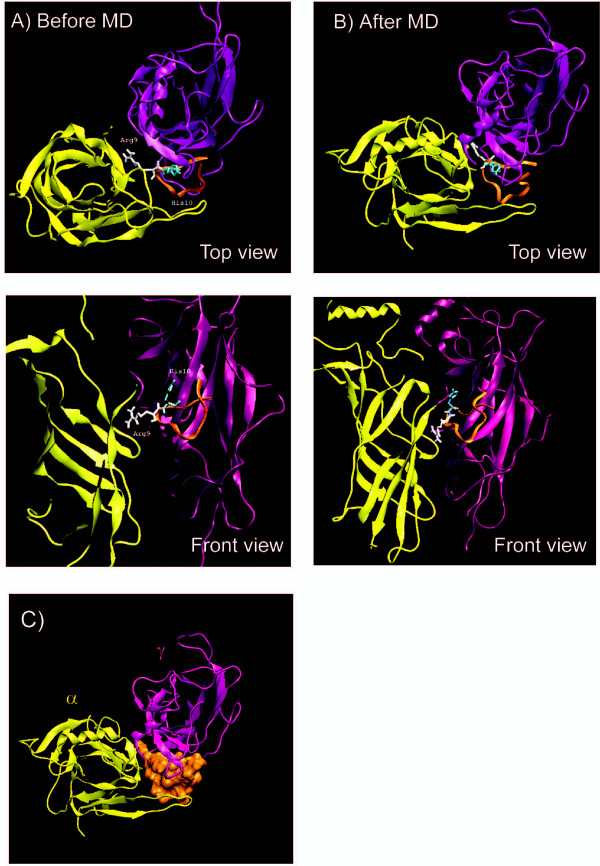
**Position of residues His10 and Arg9 in 1XGA relative to the alpha and gamma subunits (A) before and (B) after MD simulations**. (C) Protruding surface of 1XGA between the two subunits, top view.

Generally, α-conotoxin GI shows higher affinity for *Torpedo *nAChRs in the α/γ site [7]. Our docking results confirm this, and filtering the docked complexes according to the known interacting residues in the α/γ and α/δ interfaces (see Methods section) yielded apparently more "correct" results for the α/γ binding site (see Fig. 1D–E). The complexes were checked in a viewer for proper position of the ligand in the binding pocket, as well as the position of the nitrogen atom in the aromatic cage; and eventually a complex of 1XGA bound to the α/γ interface with optimum interaction energy was chosen for further analysis. Table 2 shows some of interacting residues involved in the binding of α-conotoxin 1XGA to the alpha and gamma subunits of *T*.nAChR. It has been shown that the binding sites for α-bungarotoxin and α-conotoxins only partially overlap [34]. Comparison of the regions involved in the interactions with α-neurotoxin NTX1 (table 1) and α-conotoxin 1XGA (table 2) within *T*.nAChR confirms the claim; it is obvious that certain regions such as residues 180–200, 91–94 in the alpha_1 _and some residues like Leu118 and Trp54 in the γ subunit are important for binding to these antagonists. Mutational analysis has already revealed that the positive charge of His10 is not essential while that of Arg9 is essential; the side chains of Arg9 and His10 project prominently away from the structure [7]. This protruded surface acts as the recognition site for the α/γ binding site, and it has been proposed that the region around position 9 in the α-conotoxins is oriented toward the δ and γ subunits of the nicotinic receptor [7]. Fig. 2 shows the positions of side chains Arg9 and His10 in α-conotoxin 1XGA relative to the alpha and gamma subunits in *T*.nAChR; as can be seen in Fig. 2C, the protrusion of the conotoxin molecule is located exactly in the interface between the subunits. Table 2 summarizes the main contacts between conotoxin 1XGA and subunits alpha_1_/gamma. Comparing this to the α-neurotoxin NTX-1 contacts at the very same interface, it is not unreasonable to presume that the side chains of Arg 9 and Arg 35 in conotoxin and snake neurotoxin molecules, respectively, mainly contact via hydrophobic and cation-π interactions (see tables 1 and 2). Even though long chain α-neurotoxins make multiple contact points with the receptor (mentioned earlier) and it has been shown that their binding site only partly overlaps with that of α-conotoxins, the relative similarity seen between their counterparts located in the alpha and gamma subunits is notable: Trp149 and region 90–94 in the alpha subunit and Leu118, Trp54 and Asp176 in the gamma subunit are among the common residues making contacts with the "main" parts of both toxin molecules.

**Table 2:** Amino acid residues participating in interactions of conotoxin 1XGA with the α/γ receptor interface determined by docking simulation, as well as average occupancies of the main anchor point residues participating in interactions after 7 ns molecular dynamics simulation along the trajectory.

	***T*.nAChR subunit interface**		
	
**1-XGA**	Principal site (alpha 1)	Complementary site (gamma)	Average occupancy during MD
GLU 1			
CYS 2			
CYS 3			
ASN 4	Thr 191, Cys 192		Asn4 Thr191 0.9
			Asn4 Cys192 0.8
PRO 5			
ALA 6	Tyr 190, Val 188		Ala 6 Tyr190 0.5
			Ala 6 Val 188 0.4
CYS 7	Tyr 190		Cys 7 Tyr 190 0.8
GLY 8		Trp 54	
ARG 9*	Trp 149, Val 91, Leu 92, Asp 99, Tyr 198	Leu 118, Trp 54, Thr 37, Asn 38	Arg9* Trp 149 0.7
			Arg9* Leu 92 0.9
			Arg9* Trp 54 0.9
			Arg9* Thr 37 0.8
			Arg9* Asn 38 0.8
HIS 10		Leu 118, Tyr 116, Leu 108, Arg 78	His10 Leu108 0.8
			His10 Arg 78 0.8
TYR 11		Trp 54, Thr 35, Asp 176	Tyr11 Trp54 0.9
			Tyr 11Asp 176 0.7
SER 12		Glu 56, Leu 118, Lys 33	Ser 12 Leu118 0.4
CYS 13		Tyr 116	Cys13 Tyr116 0.5

The critical residue Arg 9 is marked with an asterix. Amino acids making hydrogen bonds are underlined.

### Motions in the LBD of nAChR

#### Structure stability and fluctuations

**Figure 3:**
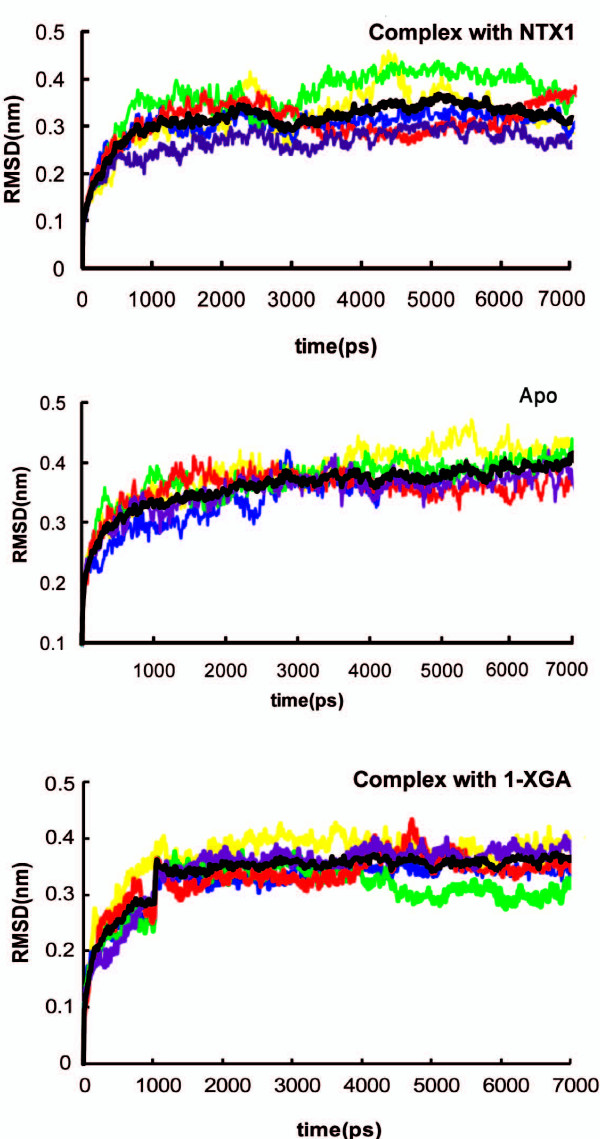
**The RMSD of all subunits and the average in apo and in complex forms of the LBD**. The color represents each subunit as in Figure 1: α_1_, β, δ, α_2 _and γ are colored yellow, blue, green, red and violet, respectively. The average of all subunits is shown in black.

The root mean square deviation (RMSD) is an indicator of the structural stability of a protein in a given environment. The RMSD plot of the C_α _atoms in nAChR in the apo (ligand-free) form and in complex with toxin molecules as a function of time for all subunits, and the average structure relative to the starting model, are shown in Fig. 3. The RMSD increases rapidly at first owing to thermal vibrations and improper interactions; the deviations seen are not unexpected for a system of this size. Comparison of the RMSD plots for the apo and complex forms shows no marked differences between these two structures; however, the complex form of the LBD with α-neurotoxin seems to be slightly stabilized. Discarding the first 1500 ps of the MD trajectory (the time needed for the system to become relatively stable), detailed analysis of the LBD motions will give important information about the structure-function relationship [see Supplementary figure 6 in Additional file 2]. It seems that upon binding of the α-neurotoxin NTX-1, the relative stability of the lower third of all subunits in the LBD increases substantially, the only exception being the second binding site (alpha_2_/delta) where the C-loop and Cys loop show more intense fluctuations. On the other hand, RMSD plots of the C-loop, F-loop and Cys loop of the LBD of the receptor in complex with α-conotoxin 1XGA shows local rigidity only within some subunits and it seems that binding of conotoxin 1XGA, because it is smaller, does not confer the same effects at least during the very early stages of binding. When the conotoxin binds, the stability of the C-loop decreases in subunits alpha_1_, beta, and to a lesser extent gamma. While RMSD of the C-loop in all subunits in complex form indicates a more stabilized local arrangement, the Cys-loop and F-loop show more deviations compared to the apo form of LBD; this is specifically seen in the case of the two alpha subunits. Here again, the decrease in local fluctuations in the C-loops is somehow concerted with an increase in movements in other important loops such as the Cys- and F-loops [see Supplementary figure 7 in additional file 3].

**Figure 4:**
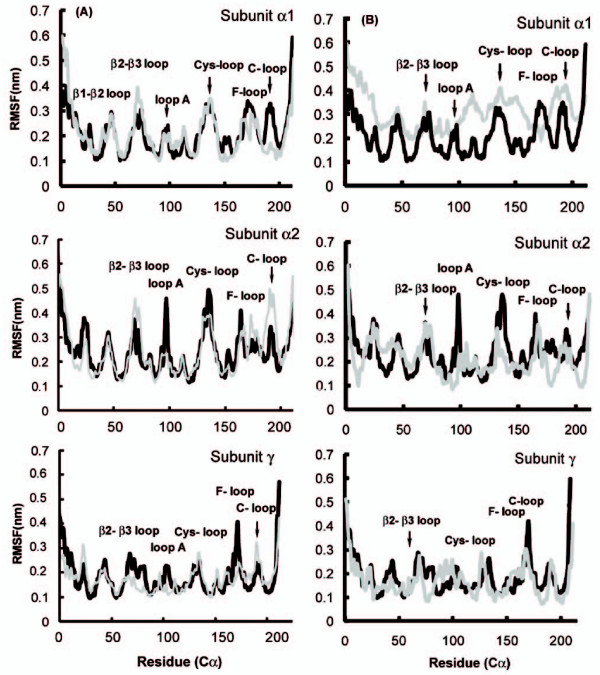
**Root mean square fluctuation (RMSF) of the apo form of LBD (*black*) and the complex (*gray*) relative to their average structure in the NTX1-LBD complex (A)**. RMSF of α and γ subunits in apo (*black*) and complex forms (*gray*) for 1XGA-LBD complex (B).

A Root-mean square fluctuation (RMSF) provides more detailed information on the mobilities of residues relative to the average structure. The RMSF of the C_α _atoms, and details of the RMSF of subunit α in apo form and as a complex of the LBD with toxin molecules, are shown in Fig. 4. Excluding the chain terminus, the mobile parts of the apo structure lie in the helix 3–13 and loops 23–26 (β1–β2 loop), 62-74 (β2–β3 loop), C-loop, Loop A, Cys loops and F-loop (Fig. 4). For the NTX1-LBD complex form, these fluctuations become more intense in the C-loops in subunits alpha_2_, delta and gamma, and the F-loop in alpha_2_; but they are restricted in the C-loop of α_1_, F-loop in gamma, Cys loop and loop A in alpha_2_, and Cys and F-loop in delta (Fig. 4A). It seems that the increase in fluctuations in C-loop regions is concerted with decreased fluctuations in the Cys loop among subunits. There is an explanation for the decreased fluctuations in the C-loop in subunit alpha_1 _and the F-loop in gamma: these are the regions directly involved in interaction with a bulky toxin molecule, therefore the motions of residues there are more restricted.

An interesting feature of the 1XGA-LBD complex is the local increase in the RMSF of subunit alpha_1 _relative to the apo form and other subunits (see Fig. 4B). This behavior was not observed in any of the other simulations; the reason for it seems to be a local perturbation caused by the presence of the ligand in the binding pocket in the early stages. Conotoxin 1XGA is smaller than α-neurotoxin NTX-1, with obviously fewer multi-point contacts and a much less flexible structure, which probably could not hinder fluctuations in this region.

### Binding site changes and subunit interfaces

#### Movement of the C-loop

Given the different natures of the component subunits in heteromeric nAChRs, it would be expected that different structural changes take place in binding sites and at the subunit interfaces. It is suggested that the two alpha subunits have an "open" arrangement in the resting state of *T*.nAChR (2BG9) [3], making the ligand-binding pocket accessible to the agonist/competitive antagonist binding. An important motion in the simulation takes place in the C-loop region that forms one side of the binding site (main side). In order to measure the movement of the C-loop in the binding pocket, the distances between the C_α_s of residues Cys 192 at the tip of the C-loop in alpha subunits, and Pro119 in complementary subunit gamma/Pro121 in delta (and similar positions in other non-alpha subunits), were measured during the 7 nanosecond simulations [see Supplementary figure 8 in additional file 4]. An interesting observation in the binding site is the "flapping" of the C-loop in the two alpha subunits in the apo form; this distance is usually greater for subunits alpha_1 _and alpha_2 _in 2BG9. The C-loop in alpha subunits remains "opened" during the 7 ns simulation, hovering around 20 Å; while in beta, gamma and delta the "lid" tends to close the binding pocket. Similar results have also been observed by others [15,17], confirming that the C-loop is mobile in apo conformations and does actually swing out. C-loop movement is apparently limited in alpha_1 _owing to the presence of the bulky toxin molecules in the binding site; the interactions with binding site and C-loop (discussed earlier) therefore restrict movements in this region (Fig. 4). In the alpha_2 _subunit in the LBD-NTX1 complex, the distance increases constantly and the C-loop continues to move outward. It can be seen that when the toxin binds in one of the two binding sites known for the competitive antagonist, the overall movement of the C-loop during the 7 ns MD simulation in alpha_2 _and beta is towards a more "open" position, making the second binding site more accessible to ligand binding; while in alpha_1_, delta and gamma it tends to move inward and form a more closely packed pocket. The situation is almost the same for the 1XGA-LBD complex; the only difference seen here is that the C-loop movement in the alpha_2 _subunit behaves like alpha_1 _and is clearly limited compared to the apo form. In the case of subunits delta and gamma, again the lid tends to close during the simulation.

### Local rearrangement associated with α-neurotoxin binding

As mentioned earlier, there are networks of loops at the interface between the LBD and TMD that couple ligand binding to channel gating. Domains C (C-loop) and F (F-loop) are two main constituents of the binding site, and of these, the C-loop exhibits more obvious displacement upon binding of agonists or competitive antagonists. The Cys-loop is another pivotal point in coupling binding to gating, since it is located at the interface between the LBD and the M2–M3 loop in TMD. Therefore, a closer look at the interactions within these locations could lead us to the initial mechanisms that transfer local changes in the binding site to the pore domain.

#### Interactions among the K145, D200 and Y190 triad

**Figure 5:**
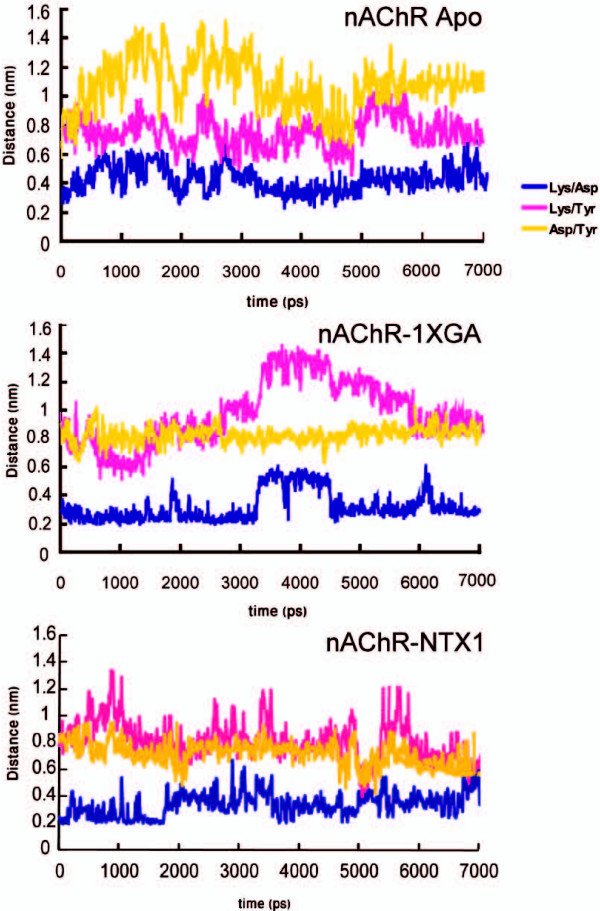
**Distance between triads Lys 145, Asp 200 and Tyr 190 in apo and complex forms**. The colors indicate each pair interaction: blue for Lys 145-Asp 200, magenta for Lys 145-Tyr 190 and yellow for Asp 200-Tyr190.

One such interaction is through salt bridges formed between Arg 206 and Glu 45, located at the bottom of the LBD, which is then transferred to the transmembrane helices M2–M3 [3,35]. It has not been possible to study this currently owing to the absence of the transmembrane domain. The other important intra-subunit interaction is between three conserved residues at the main ACh binding site (α subunit): Lys 145, Asp 200 and Tyr 190. Kinetic analysis and site-directed mutagenesis, as well as structural modeling of AChBP with ligand-bound (carbamylcholine) and ligand-free conformations, have recently shown that local rearrangements associated with agonist binding propagate through beta strands to the pore domain [35-37]. By analogy with the AChBP in the resting state of the muscle type nAChR, K145 and D200 in the C-loop are proposed to pair through electrostatic forces, and Y190 is out of register. When the agonist binds, Y190 is drawn towards the K145/D200 pair and pulls K145 away from D200 [36]. These local displacements could propagate to the channel via β-strands 7 (harboring K145) and 10 (harboring D200), which are linked to the M2–M3 and M1 transmembrane domain respectively. Among these three residues, Y190 is directly linked to the ACh binding site. It is located in the aromatic cage surrounding the positively-charged agonist; in the case of α-neurotoxins as competitive antagonists, Arg36 (Arg35 in NTX-1) and Arg9 in α-conotoxin 1XGA play the role of positively-charged agonist. In our complex model, Tyr190, located at the C-loop, actually moves about 3.5 Å towards Lys145; it was shown previously that in the complex form, the C-loop is drawn slightly towards the binding pocket after 7 ns MD simulation. Nevertheless, this movement has little or no effect on the interaction between Lys145 and Asp200 during the simulation. When the toxin binds, it is clearly seen that Lys145 and Asp200 have faced towards each other after 7 ns MD simulation, making the distance between these residues even less relative to the apo form (see Fig. 5). This decrease in distance stabilizes the electrostatic force between the residues, so the salt bridge between K145/D200 pairs not only remains intact but also strengthens; while in the apo form of the receptor this distance is variable, making the salt bridge more labile than in the complex form (see Fig. 5). A possible explanation for this is that in the ligand-free (apo) form, the intense fluctuations of the C-loop make Tyr190 – located at the tip of the C-loop – fluctuate in its place, often moving towards the K145/D200 pair. This fluctuation would form transient electrostatic interactions between Y190 and the K145, which could have an effect on the salt bridge between K145/D200 and weaken the electrostatic bond. On the other hand, when the toxin binds, Tyr190 is extensively involved in a hydrophobic interaction with Trp31 after about two nanoseconds (not shown) and also a transient hydrogen bond with Arg35 [see Supplementary table 3 in additional file 1]. These interactions would largely restrict the Tyr190 fluctuations, leaving the K145/D200 pair alone. In addition, because the toxin molecule is bulky (compared to small agonists such as ACh or CCh) the C-loop (or the "lid") is not closed completely and in fact remains opened, therefore Tyr190 is never close enough to K145 to disrupt its salt bridge with D200 effectively. Since C-loop displacement would propagate to the pore via β-strands 7 and 10 [see fig. 9 in additional file 5] as an initial link to the gating cascade, it can be proposed that restricting the motions in C-loops when an α-neurotoxin binds would prevent the large displacements of the C-loop that ultimately results in channel opening. This is in agreement with the assumption that binding of α-neurotoxin stabilizes the "resting" or basal state of the receptor, while the whole LBD without toxin bound to it seems to move towards a more relaxed state or at least an intermediate conformation between the open and resting states.

In the conotoxin-LBD complex, Tyr190 is extensively involved in hydrogen bonding with Tyr198 (alpha_1_) and a non-bonded interaction with Cys7 (1XGA) during the simulation (see table 2). The C-loop in the conotoxin-LBD complex does not swing during the simulation [see Supplementary figure 7 in additional file 3], so the residue Tyr190 at the tip of the C-loop keeps its contacts during the simulation with the ligand and also with Tyr198; Tyr190 would therefore not disrupt the salt bridge between the K145/D200 pair. Fig. 5 shows that the contact between the K145/Asp200 pair is likely to be more stable than in the apo form; also, because Tyr190 is partially "fixed", the other contacts that depend on Tyr190 are much more stabilized. It seems that upon binding of relatively large antagonists, the C-loop, which plays the major role in transferring the local changes to the transmembrane domain, is extensively engaged in multiple contacts with these ligands, which as a consequence substantially hinder its displacements.

## Conclusion

Advances in drug discovery/drug design focusing on new selective nAChR compounds are due to comprehensive studies of their structures. Agonist or antagonist drugs that selectively target receptor subtypes could be designed to maximize the desired effect and minimize side-effects. Toxins bind with higher affinity than endogenous ligands; therefore an accurate prediction of their binding mode can inform the design of more efficient lead compounds. Details of interactions with subtype-selective antagonists such as α-conotoxins and α-neurotoxins may prove beneficial in the treatment of certain neuropathologies and diseases.

In the current study, the interaction and dynamics of a long-chain α-neurotoxin and an α-conotoxin with *T*.nAChR have been studied over a 7 nanosecond molecular dynamics simulation. The proposed model has shown to accord with numerous experimental data, proving the validity of the model and the methodology. Our docking results confirm the claim that *T*.nAChR (2BG9) is in a "basal" or "resting" state that favors binding to α-neurotoxins; this has been reported for α-cobratoxin in another study [20]. Our results also confirm that the asymmetric "resting" state of the LBD in nAChR has a dynamic nature. Each subunit behaves specifically and individually, which is a result of the hetero-pentameric conformation of the receptor.

The formation of an aromatic cage upon agonist (ACh) binding is believed to be one of the steps in the allosteric pathway and α-neurotoxins are proposed to occupy and even partially overlap the ACh binding site, so they are deemed competitive antagonists. Our model supports the fact that Arg35 at the tip of loop II in the NTX-1 mimics the behavior of ACh: it is surrounded by W149, Y93 and Y190 from the main side (α_1_subunit) and W54, L118 from the complementary side (γ subunit). Similar interactions are seen for Arg9 in conotoxin 1XGA: this is also surrounded by the aromatic residues W149, L92, Y198 from the α subunit and W54, L118 from γ. The Arg9 residue in α-conotoxin GI plays a key role in the interaction with the high affinity binding site in *T*.nAChR [38], hence indicating that its counterparts in the α/γ interface would be useful in designing more specific nAChR ligands.

Upon binding of the toxins, local rearrangements are induced in the binding site. Some of these are similar to what has previously been reported for small agonist binding; however, because of the bulk of the toxin molecules and their multi-point contacts with the receptor, it seems that these changes would not induce the same effects. The salt bridge formed between K145 and D200 – which should remain intact in the resting state – was labile and would weaken gradually in the apo form owing to the movements of the C-loop. However, in the complex form of NTX1-LBD, this interaction remains intact, at least during the first 7 nanosecond simulation, which would stabilize the resting state as had been previously suggested for α-neurotoxins. In the case of 1XGA-LBD this interaction is again slightly more stabilized than in the apo form. The importance of this triad is that its components are found in α_1 _subunits in all species of muscle type and most neuronal type nAChRs, suggesting a role in binding-to-gating transduction. It should be mentioned that this displacement is not the only allosteric link between binding and gating [36], but it can give a clue on how the cascade of interactions is triggered at the very early stages of binding.

## Methods

### Homology modeling

Models of the extracellular domain of *T*.marmorata nAChR subunits were constructed using the program MODELLER 9v1 [39]. Missing F-loop residues were replaced and the F-loops were modeled again using the "model-loop" command. Each subunit was constructed separately. Beta, delta and gamma subunits were constructed using the B, C and E chains of *T*.marmorata nAChR with substitution of 9, 10 and 7 residues respectively. The subunits were then assembled into pentamers using the same rotational angles in 2BG9. The modeled structure was energy minimized using GROMOS96 implementations included in the Swiss-pdb Viewer (version 3.7) [40].

### Docking simulation

Docking was performed using the 3D-Dock suite [41,42]. The α-neurotoxin NTX-1 structures were chosen as snapshots from a 17 nanosecond MD trajectory (with 500 ps intervals) along with 20 NMR structures [20] as input to the program. The parameters set for the FTDock runs were as follows: global surface thickness 1.4 Ǻ, grid cell span 0.875 Ǻ and search angle step 12°. A total of 9240 rotations were evaluated, leading to 10,000 complexes for each FTDock run. The complexes were then ranked using a pair potential matrix that scores each complex according to an empirically-derived likelihood of residue contacts in a sample set of non-homologous interfaces in PDB. The resulting complexes were filtered by applying three distance constraints known to exist in the binding interfaces of ligands with subunits alpha/gamma and alpha/delta: for long-chain α-neurotoxins the known interacting residues were Arg35 in the ligand and Trp55 in the gamma subunit; Arg35 in the ligand and leu119 in the gamma subunit; Asp29 in the ligand and Tyr 190 in the alpha subunit. In the case of α-conotoxin the residues known to be involved in the interaction of conotoxin GI and MI with *T*.nAChR were selected for the filtration process [43,34]; for α-conotoxin 1XGA, all 35 NMR structures in the PDB were used for the docking procedure. The known interacting residues were Ala6, Cys7 and Arg9 and region 180–200 in the alpha subunit. The distance cut-off for intermolecular interface and non-bonded interactions was 10 Ǻ (default). All other parameters for mean-field optimization and rigid-body energy minimization in program Multidock (3D dock suite) were set as default. Subsequent visual analysis in the Swiss-pdb Viewer allowed us to reject those solutions that were not in at least rough accordance with the available biochemical and mutagenesis data for long-chain alpha neurotoxins, and in the case of α-conotoxin GI, the complex was compared to the X-ray structure of the AChBP with conotoxin IMI (PDB code:2C9T). The position of the toxin relative to each interacting subunit and its distance from the base of the LBD, as well as the positions of loops I and III relative to the top and bottom of the LBD, were checked for agreement with available experimental data for long-chain α-neurotoxins in complex with nAChR and AChBP. Proper complexes were found in both the α/δ and α/γ interfaces, from which the solution in the alpha-gamma interface (high affinity binding site for these antagonists) was selected and subjected to the molecular dynamics simulation.

### Molecular dynamics (MD)

Further energy minimization and molecular dynamics were carried out using Gromacs 3.3.1 [44-46] for the complex and ligand-binding domain (LBD) of 2BG9 free of ligand. The GROMOS96 [47] force field parameters were employed for the simulations. Periodic boundary conditions and the particle mesh ewald were used with non-bonded cut-off of 10 Ǻ, non-bonded pair list distance 10 Ǻ; and the constraint algorithm used was LINCS [48]. In each case, the system was first minimized in 500 steps using the steepest descent algorithm. The box was neutralized with the proper number of ions added and a 20 picosecond position-restrained dynamics simulation followed this, with Berendsen's temperature and pressure coupling method [49]. Each simulation was continued for a further seven (complex) and ten (LBD in apo form) nanoseconds. The data were analyzed further using the analysis commands in GROMACS, Program Ligplot [50], Spdb Viewer, and VMD [51]. Analysis of the data over the simulations was done at 10-ps intervals.

## Competing interests

The authors declare that they have no competing interests.

## Authors' contributions

This work was some of the PhD project of **Adak Nasiripourdori **and she prepared the initial draft; **Bijan Ranjbar **was supervisor of work, read and edit the manuscript and corresponding author; **Hossein N**aderi-manesh was co-supervisor of work. All authors read and approved the final manuscript.

## Supplementary Material

Additional file 1 **Table 3**. Amino acid residues participating in interactions of NTX-1 with receptor (α/γ interface) after 7 nsec molecular dynamics simulation (Gromacs 3.3.1). Click here for file

Additional file 2 **Figure 6**. (*A*) Plots of RMSD for the C-loop, Cys-loop and F-loops in Apo and complex form of the LBD with α-neurotoxin NTX1 in each subunit. Black and gray lines are used for apo and complex form, respectively. Click here for file

Additional file 3 **Figure 7**. Plots of RMSD for the C-loop, Cys-loop and F-loops in Apo and complex form of the LBD with α-conotoxin 1XGA in each subunit. Black and gray lines are used for apo and complex form, respectively. Click here for file

Additional file 4 **Figure 8**. The distance between C_α_s of Cys192 at the tip of C-loop in α1 subunit and Pro119 (γ), Asp 192 (β) and Pro 121 (α1), His 119 (β) and Phe 191 (δ), Cys 192 at the tip of C-loop in α2 subunit and Pro 122 (δ), Pro121 (α2) and Thr 192 (γ) as a function of time in (a) apo form of LBD and (b) in complex with α-neurotoxin NTX1,(c) in complex with α-cobratoxin 1XGA. The coloring is according to the principal subunits: α/γ, β/α, δ/β, α2/δ and γ/α2 interfaces are shown in yellow, blue, green, red and purple, respectively. Click here for file

Additional file 5 **Figure 9**. Side view of a nAChR promoter from outside the pentameric ring. Functional loops and beta strands are numbered according to *T*.nAChR (*ref*. [3]). Click here for file
