# Supplementary figures and images for: Binding of long-chain α-neurotoxin would stabilize the resting state of nAChR: A comparative study with α-conotoxin
Source: Theor Biol Med Model. 2009 Feb 11;6:3. doi: 10.1186/1742-4682-6-3 (PMC2649906; doi:10.1186/1742-4682-6-3)

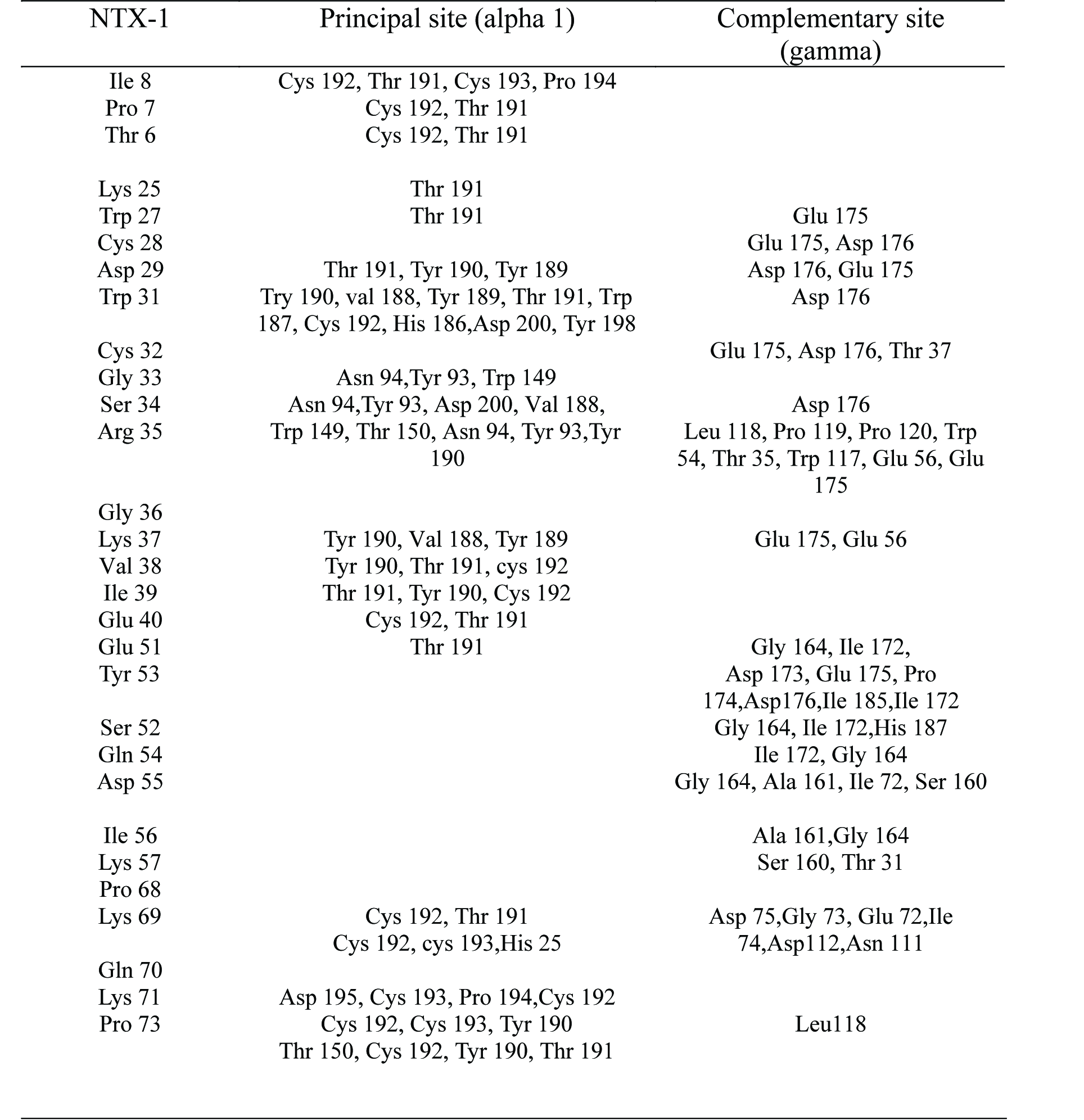

Supplement: Additional file 1 — Table 3. Amino acid residues participating in interactions of NTX-1 with receptor (α/γ interface) after 7 nsec molecular dynamics simulation (Gromacs 3.3.1). [file 1742-4682-6-3-S1.tiff]

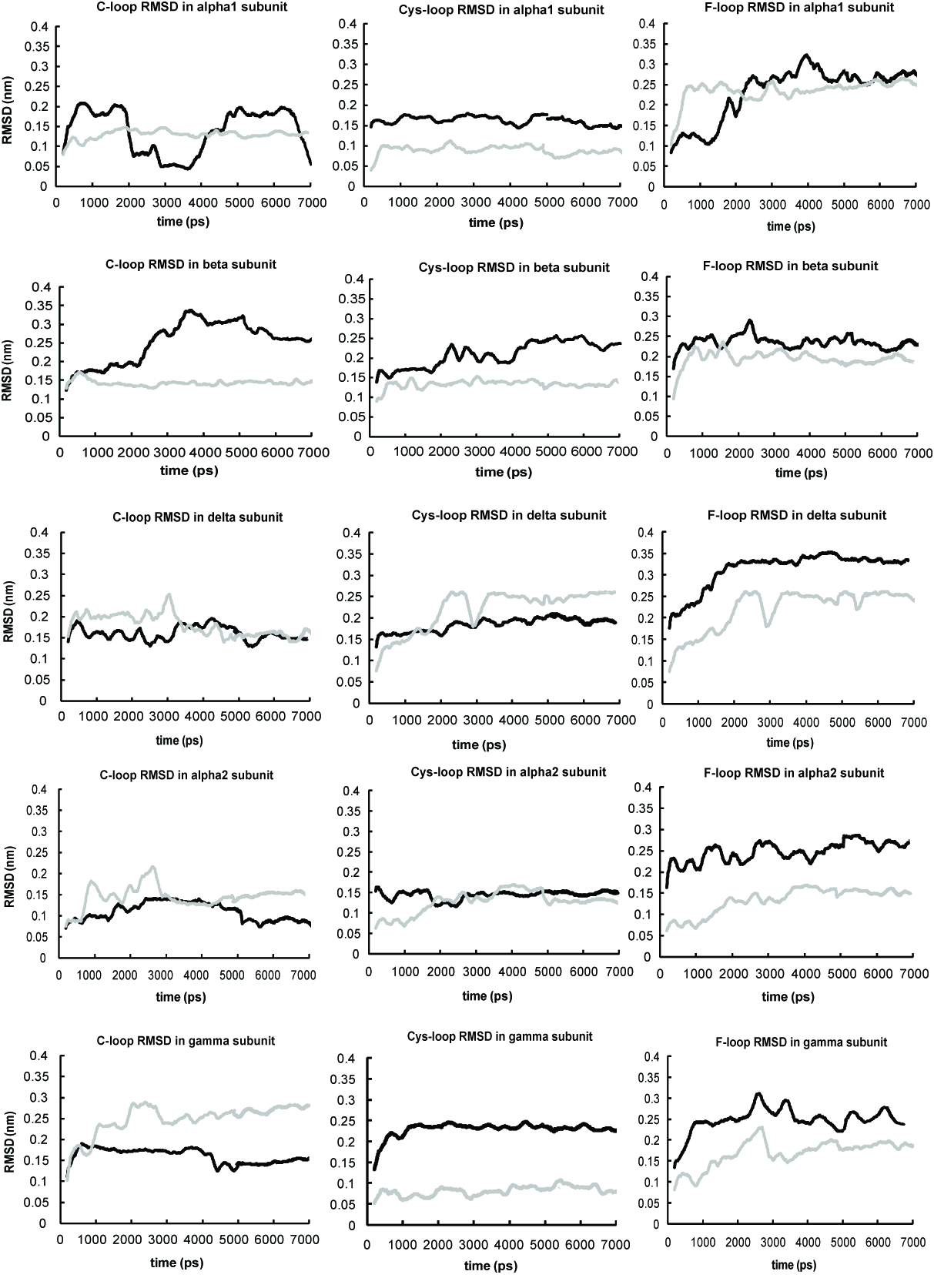

Supplement: Additional file 2 — Figure 6. (A) Plots of RMSD for the C-loop, Cys-loop and F-loops in Apo and complex form of the LBD with α-neurotoxin NTX1 in each subunit. Black and gray lines are used for apo and complex form, respectively. [file 1742-4682-6-3-S2.tiff]

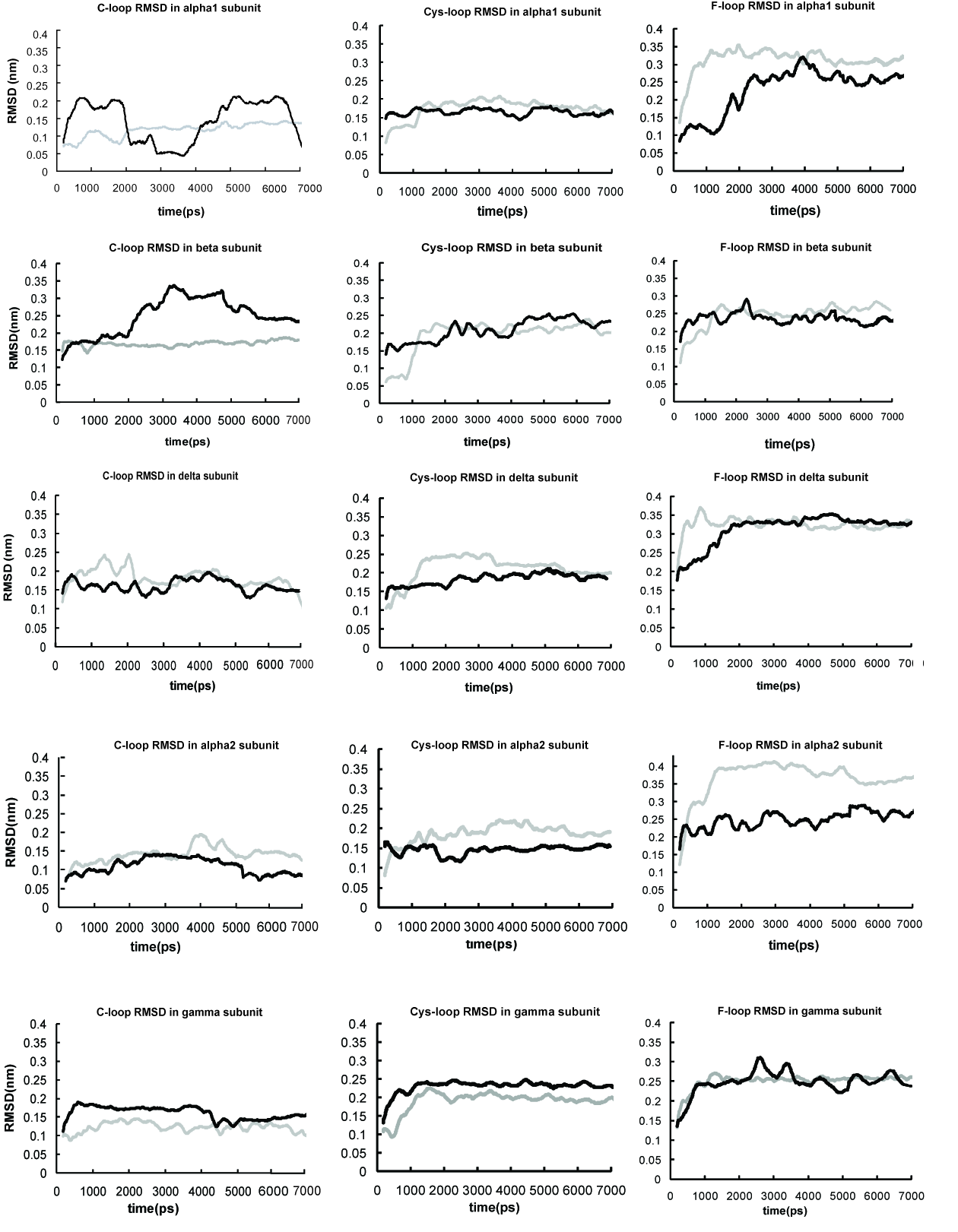

Supplement: Additional file 3 — Figure 7. Plots of RMSD for the C-loop, Cys-loop and F-loops in Apo and complex form of the LBD with α-conotoxin 1XGA in each subunit. Black and gray lines are used for apo and complex form, respectively. [file 1742-4682-6-3-S3.tiff]

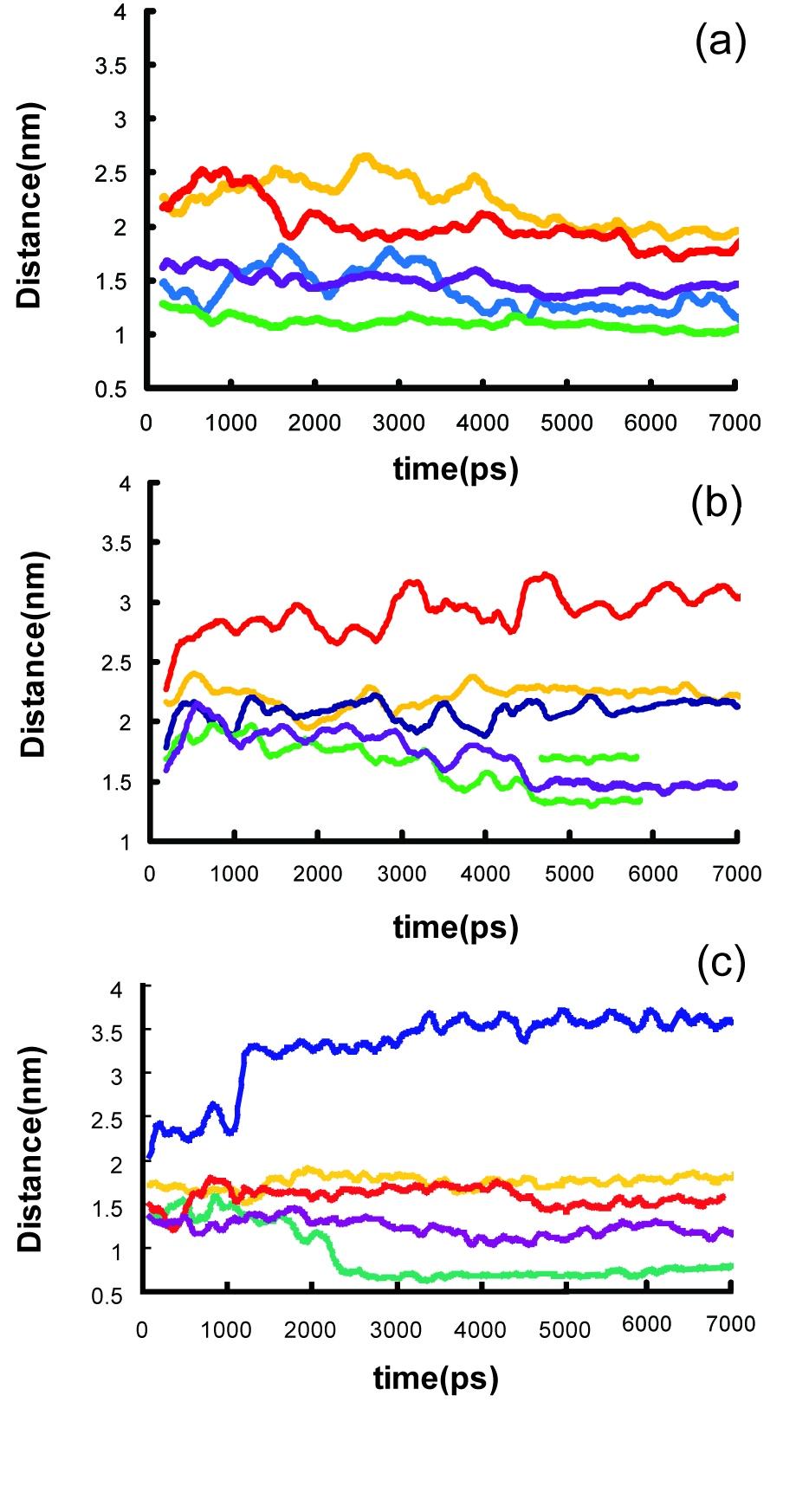

Supplement: Additional file 4 — Figure 8. The distance between Cαs of Cys192 at the tip of C-loop in α1 subunit and Pro119 (γ), Asp 192 (β) and Pro 121 (α1), His 119 (β) and Phe 191 (δ), Cys 192 at the tip of C-loop in α2 subunit and Pro 122 (δ), Pro121 (α2) and Thr 192 (γ) as a function of time in (a) apo form of LBD and (b) in complex with α-neurotoxin NTX1,(c) in complex with α-cobratoxin 1XGA. The coloring is according to the principal subunits: α/γ, β/α, δ/β, α2/δ and γ/α2 interfaces are shown in yellow, blue, green, red and purple, respectively. [file 1742-4682-6-3-S4.tiff]

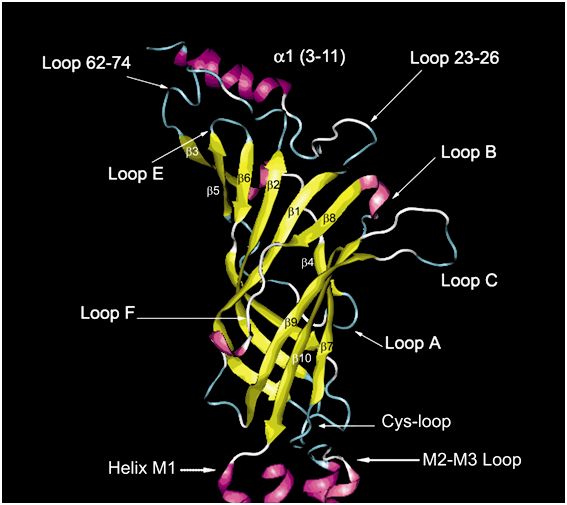

Supplement: Additional file 5 — Figure 9. Side view of a nAChR promoter from outside the pentameric ring. Functional loops and beta strands are numbered according to T.nAChR (ref. [3]). [file 1742-4682-6-3-S5.tiff]
